# Effect of Nebulized Furosemide on the Mortality of Adult, Mechanically Ventilated Acute Respiratory Distress Syndrome (ARDS) Patients: Protocol of a Randomized Clinical Trial (The ENHALE Trial)

**DOI:** 10.7759/cureus.81006

**Published:** 2025-03-22

**Authors:** Ahmed F Mady, Hend M Hamido, Basheer Abdulrahman, Anas A Mady, Ahmed W Aletreby, Ahmed A Abdalla, Jennifer Q Gano, Waleed T Hashim Aletreby

**Affiliations:** 1 Anesthesiology and Intensive Care, Tanta University Hospitals, Tanta, EGY; 2 Critical Care Medicine, King Saud Medical City, Riyadh, SAU; 3 College of Medicine, Alfaisal University College of Medicine, Riyadh, SAU; 4 Faculty of Medicine, Alexandria University, Alexandria, EGY

**Keywords:** acute respiratory distress syndrome (ards), furosemide nebulisation, mechanical vent, medical icu, mortality

## Abstract

Background

Acute respiratory distress syndrome (ARDS) affects a significant proportion of ICU patients and has a high mortality rate. The inflammation of the alveolar-capillary membrane is pathognomonic and characterized by increased capillary permeability and pulmonary edema. A safe, readily available anti-inflammatory agent delivered directly to the lungs could be a promising therapeutic approach. All these properties apply to nebulized furosemide.

Objectives

The primary objective of this study is to analyze 28-day all-cause ICU mortality while the secondary objectives include assessing hospital mortality, ICU and hospital length of stay (LOS), ventilator-free days at 28 days, successful extubation rate, and adverse events.

Methods

A double-blind, placebo-controlled, parallel-arm superiority RCT using an intention-to-treat (ITT) analysis to assess whether nebulized furosemide reduces mortality in adult mechanically ventilated ARDS patients will be employed.

Results

The results of descriptive and inferential analyses will be tabulated, and the significant results will be presented.

## Introduction

Acute respiratory distress syndrome (ARDS) is usually defined as rapidly evolving hypoxemia due to pulmonary edema originating from causes other than cardiogenic such as increased alveolar-capillary permeability [1,2]. The Berlin Definition, established in 2012 by an expert panel, provides an objective clinical framework for defining ARDS [1]. ARDS encompasses a wide spectrum of risk factors and/or causes that could be generally divided into direct and indirect causes [3], direct causes may include aspiration, pneumonia, lung contusion, inhalational injury, and near drowning [4], whereas examples of indirect causes include sepsis, transfusion, hemorrhagic shock [4], pancreatitis, burns, drugs, or toxins [5]. Some studies have also associated tobacco and alcohol use, hypoalbuminemia, air pollution, and recent chemotherapy with increased risk of ARDS [2,6,7].

ARDS accounts for 10% of ICU admissions and 25% of ventilated patients, affecting 3 million people annually worldwide [4,8]. The prevalence may rise with broader definitions that include high-flow nasal oxygen patients [9]. Reported mortality rates remain as high as 30%-40% or more [2,8]. This may be because of the myriad of causes and risk factors of ARDS [2], it could also be due to the difficulty in distinguishing ARDS from its risk factors as the cause of death [10], the challenging setting of ARDS management, which usually encompasses multiple organ failure, but possibly more important, due to the lack of definitive therapy, and the management being mainly supportive [11].

A recent multicenter phase II randomized controlled trial (RCT) explored the effect of nebulized furosemide in intubated COVID-19 patients [12]. The study did not reach its target sample size due to a lack of recruitment by early 2023; consequently, all of its objectives did not reach the level of statistical significance. However, there were obvious trends of the beneficial effects of nebulized furosemide. The intervention group had a higher change from day one to day six of partial pressure of oxygen in arterial blood (PaO2) to fraction of inspired oxygen (FiO2) ratio (P/F ratio), lower 60-day mortality, lower hospital length of stay (LOS), and longer ventilator-free days, with no reported adverse events.

We hypothesize that the use of nebulized furosemide in adult, mechanically ventilated patients with ARDS admitted to the ICU may decrease 28-day mortality.

Rationale of nebulized furosemide in ARDS

ARDS is characterized by an acute onset of inflammatory lung injury, with impaired gas exchange and non-compliant “stiff” lungs [13]. A histologic hallmark of ARDS is capillary endothelial injury and diffuse alveolar damage, manifesting typical inflammation, apoptosis, and necrosis of pulmonary epithelial and endothelial cells, leading to increased alveolar-capillary permeability, ultimately resulting in alveolar edema and proteinosis, and may also include alveolar hemorrhage, pulmonary capillary congestion, interstitial edema, and hyaline membrane formation [14].

An ideal therapeutic agent should possess broad anti-inflammatory activity. Several agents, such as corticosteroids and immunosuppressants, exhibit these properties. However, their systemic administration has drawbacks such as myopathy and decreased immunity, in addition to being expensive, difficult to produce, and a lack or shortage in some countries [12]. Hence, the ideal therapy would also have to be cost-effective, readily available, associated with low toxicity, and could be directly delivered to the lung tissue [12].

Furosemide (4-chloro-5-sulfamoyl-N-furfuryl-anthranilate) is a powerful loop diuretic that acts on the thick portion of the ascending limb of the loop of Henle by binding to the sodium-potassium cotransporter. It has a short half-life and low bioavailability, in addition to being inexpensive and readily available [12,15]. Nebulized furosemide has been used for a variety of lung conditions, such as dyspnea and bronchial asthma [16,17], owing to not only the presumed higher concentrations delivered to the pulmonary tissue via nebulization compared to systemic or oral routes but also due to its anti-inflammatory characteristics [12].

Furosemide is an analog of 3-hydroxyanthranilic acid (3HA) (a tryptophan metabolite). 3HA is diffusely found in the human body and can suppress T-cell responses [18], thus suppressing the production of several inflammatory cytokines [19]. In vitro, furosemide was reported to reduce the lipo-polysaccharide-induced release of inflammatory cytokines, such as interleukin-6 (IL- 6) and tumor necrotizing factor (TNF), in addition to inducing macrophage phenotypic changes from the pro-inflammatory M1 state to the anti-inflammatory M2 state and promoting the release of anti-inflammatory cytokines such as arginase [20]. The reduction of inflammatory cytokines was also reported by clinical animal studies [21]. Similarly, human clinical studies demonstrated the beneficial effect of nebulized furosemide in the treatment of pulmonary edema [15], its effectiveness in reducing pulmonary epithelial permeability in smokers, and even restoring it to normal in asthmatic patients [[22]. Hence the use of nebulized furosemide in the treatment of several lung conditions and symptoms such as bronchial asthma, dyspnea, breathlessness, and chronic obstructive pulmonary disease (COPD) in many studies, with promising results [23-26].

## Materials and methods

Study design

This is a single-center, double-blind, placebo-controlled, parallel arms, superiority randomized clinical trial. The maximum duration of the intervention will be 28 days while the duration of follow-up will be till hospital discharge. The study will be conducted in the ICU of the largest government hospital in the central region of Saudi Arabia. The ICU has 110 beds, fully equipped with invasive and non-invasive monitoring and ventilation capabilities. It is covered round the clock by intensivists, with a nurse-patient ratio of 1: 1.

Inclusion and Exclusion Criteria

Inclusion criteria: Adults (≥18 years), ICU admission, mechanical ventilation for <7 days, and ARDS diagnosed within 24 hours per the Berlin Definition [1], which includes: a) Chest x-ray showing bilateral opacities, not fully explained by effusions, lobar/lung collapse, or nodules; b) Respiratory failure not fully explained by cardiac failure or fluid overload, and exclusion of hydrostatic edema (by echocardiography); c) Oxygenation and ventilator settings matching one of the three categories of ARDS: Mild: 200 mmHg < PaO2/FIO2 ≤ 300 mmHg with positive end-expiratory pressure (PEEP) or continuous positive airway pressure (CPAP) ≥ 5 cm H2O. Moderate: 100 mm Hg < PaO2/FIO2 ≤ 200 mm Hg with PEEP ≥ 5 cm H2O. Severe: PaO2/FIO2 ≤ 100 mm Hg with PEEP ≥ 5 cm H2O.

Exclusion criteria: Pregnant or lactating ladies; Patients who are not expected to survive more than 48 hours, according to the treating team; Mechanical ventilation is expected to continue for less than 48 hours (due to rapid recovery) according to the treating team; Advanced directive of Do Not Resuscitate (DNR); Refusal to participate in the trial by the patient or official surrogate; Known allergy to furosemide; Previous enrollment in the trial (a patient can only be enrolled once).

Study objectives

The primary objective is 28-day mortality in the ICU, starting from the day of randomization. Secondary objectives include: All-cause hospital mortality, ICU and hospital length of stay (LOS), the difference in P/F ratio between day one and day seven (not to be calculated for patients who are censored before seven days), 28 days ventilator-free days (VFD) (defined as days without mechanical ventilation till day 28 starting from day of enrollment, as long as the patient is alive); patients still mechanically ventilated at day 28, or died by day 28, will be assigned zero VFD. If the artificial airway is shifted from endotracheal tube to tracheostomy, they will still be considered mechanically ventilated until they are liberated from mechanical ventilation and are spontaneously breathing. With respect to a successful extubation rate, defined as extubated and not requiring re-intubation for 72 hours, tracheostomy is not considered successful extubation. Examples of adverse events will be hypersensitivity reactions - of any magnitude, abnormal lab investigations, specifically electrolytes and renal function tests, and volume of urine output.

Enrollment, randomization, and allocation concealment

After obtaining the informed consent of a legal guardian, randomization will be obtained via a phone call providing a study code, without breaking the concealment of allocation. Randomization will be performed using variable block sizes (4, 6, or 8), stratified by ARDS severity (mild, moderate, severe).

Blinding

**Figure 1:**
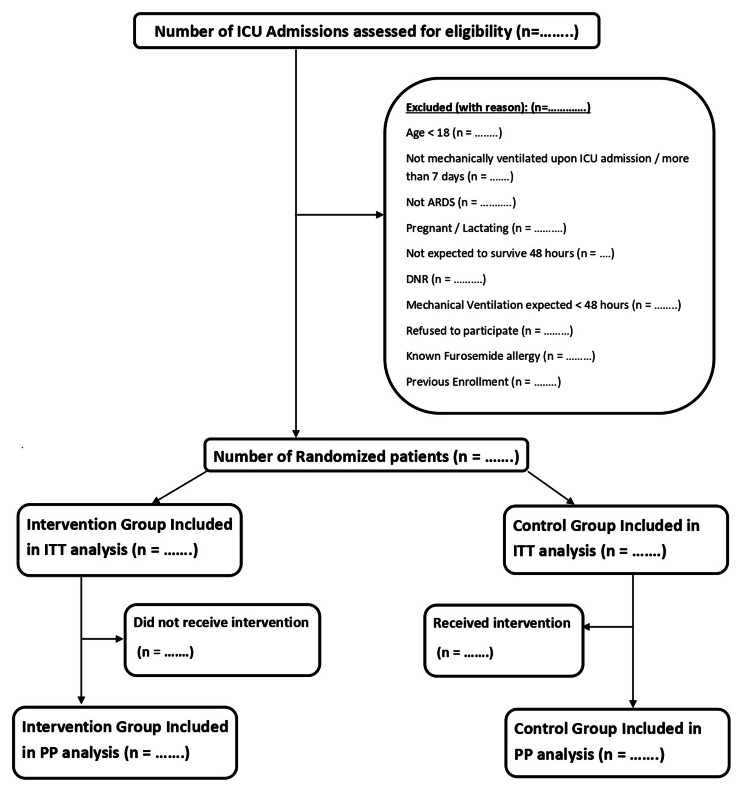
Flowchart of patients' enrollment ITT = Intention to Treat, PP = Per Protocol

Furosemide and saline are identical in appearance, color, and solution characteristics. The investigational medication will be prepared by an independent pharmacist in identical vials, labeled only with study code and patient identifiers, and delivered to the clinical area. The flow of patients' enrollment is depicted in Figure 1.

Study intervention

The study intervention should begin within six hours of randomization. Identical vials of 4 ml containing either furosemide (intervention) or 0.9% saline (placebo). In this study, we will follow a nebulized furosemide protocol similar to that reported in a study on nebulized furosemide in COVID-19 patients [12].

The intervention medication is 40 mg furosemide in 4 ml of 0.9% saline, administered every six hours via the ventilator circuit over 30 minutes. The nebulization circuit used in our ICU is Aerogen® (Galway, Ireland). The intervention continues till extubation, death, or completion of 28 days, whichever comes first. The intervention will continue if the artificial airway is shifted from the endotracheal tube to tracheostomy but not for re-intubated patients.

The control group receives a similar color and volume of 0.9% saline following the same intervals, route, and duration.

Common management

This is a pragmatic trial; the study protocol does not control every aspect of the management, apart from the intervention medication, trying to resemble real-life scenarios of management, where different treating teams may have slightly different methods of management, which may provide higher external validity [27].

However, our center follows evidence-based guidelines and protocols for the management of ARDS [28], and treating teams will be encouraged to follow those guidelines such as protective lung strategy. Adjuvant and rescue therapies will be allowed, such as (but not limited to) prone positioning and extracorporeal membrane oxygenation (ECMO), and the use of other medications such as glucocorticoids, antibiotics, and neuromuscular blocking agents.

Similarly, our center adopts practices of early enteral feeding, light sedation, and daily sedation vacation when applicable, deep vein thrombosis (DVT) and gastrointestinal (GIT) prophylaxis, and early weaning and extubation using a standardized protocol. Antibiotics are prescribed after consulting infectious disease specialists.

Sample size calculation

Assuming a mortality rate of 40% in the control group [2,8], and aiming to detect an effect size of 10% absolute reduction in mortality in the intervention group with a power of 80% and type I error rate of 5%, we estimated the sample size to be 712 patients (356 in each group), with 10% inflation to compensate for possible loss of follow-up (patients transferred to other healthcare facilities), we plan to recruit a total of 784 patients, allocated in a 1: 1 ratio as 392 patients in each group.

Statistical plan

Intention-to-treat (ITT)-based analysis: Data of the patients will be analyzed in the group to which they were randomized, regardless of the actual intervention they received. Continuous variables will be summarized as mean ± standard deviation (SD) or median and interquartile range (IQR) depending on the normality assumption, tested for by the Shapiro-Wilk normality test. Discrete variables will be summarized as frequency (count) and percentage. Time-to-event data will be 28-day mortality as the event and duration since randomization as the time variable, with right censoring.

For the primary outcome, comparison between both groups for 28-day mortality will take place by the chi-square test of association, for secondary outcomes, groups will be compared by the chi-square test of association or Fisher’s exact test, according to data count in each cell of the two by two contingency table, while continuous variables will be compared between groups by the student's t-test or the non-parametric Wilcoxon Rank-Sum test.

We will perform a survival analysis using the time-to-event data, in the form of a Kaplan-Meier Survival curve as compared by the log-rank test.

As a sensitivity test for the primary outcome, we will fit two regression models. First will be a multinomial logistic regression model, using backward elimination to retain only variables with p-value < 0.1 and the severity of ARDS as the multinomial levels in the model. A multivariable proportional hazard model (Cox regression) will be carried out in a similar way, utilizing days from randomization to event as the time variable. Additionally, we will present a per-protocol analysis of the primary outcome.

A priori subgroup analyses will examine the primary outcome with regard to sex, the median age of the cohort, ARDS severity, and adjunct and rescue therapies used (such as corticosteroids, prone positioning, and ECMO).

There will be no correction for multiple testing; accordingly, results of secondary outcomes and sub-group analyses should not be considered conclusive and must be cautiously interpreted.

All statistical tests are two-sided and considered statistically significant if the p-value is <0.05.

Interim analysis and stopping rules

**Figure 2:**
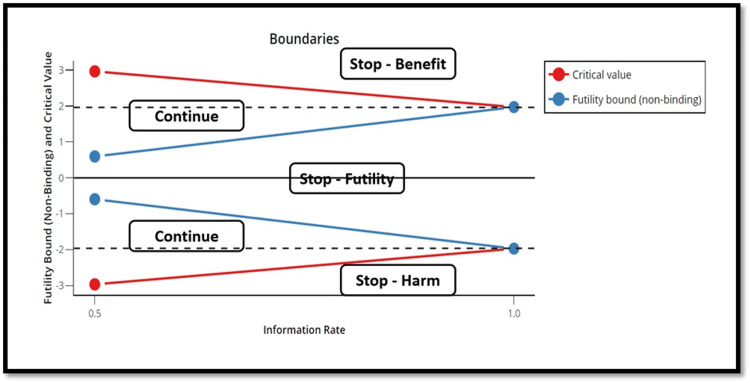
Trial stopping bounds The trial may be stopped at the first interim analysis for harm or benefit (p = 0.0031) or futility (p = 0.55).

We will perform one interim analysis when 50% of the sample size is recruited, to assess the efficacy of the intervention, with the possible early stopping of the trial based on O’Brien-Fleming alpha spending functions [29]. The stopping rules are: 1- Stopping for superiority: If the interim analysis results in a critical z-score of +2.963 or more, yielding a p-value of 0.0031 or less; 2- Stopping for harm: If the interim analysis results in a critical z-score of -2.963 or less, yielding a p-value of 0.0031 or less; 3- Stopping for futility: If the interim analysis results in a critical z-score between +0.595 and -0.595, corresponding to a p-value of 0.55 (Figure 2).

Ethical considerations

This protocol was approved by the institutional review board of King Saud Medical City (Riyadh, Saudi Arabia) (reference number: H1RI-19-Aug24-01). The duration of the initial IRB approval was three months and this is extendable after we present a progress report indicating that the trial is still ongoing. The protocol was registered with the ISRCTN registry (registration number ISRCTN16516753; dated March 23, 2025). The study will abide by the general rules of research subject protection outlined by the Declaration of Helsinki.

## Results

All results of group comparisons will be presented with either mean or percent difference, along with the corresponding 95% confidence interval (CI). The main results will be presented within the text of the results section while all results will be presented in table formats; the first dummy table outlines the baseline characteristics of the enrolled patients and the second dummy table will include the outcomes (dummy Tables 1, 2).

**Table 1:** Dummy table showing baseline characteristics SOFA = Sequential Organ Failure Assessment, CFS = Clinical Frailty Scale, BMI = Body Mass index, CKD = Chronic Kidney Disease, CLD = Chronic Liver Disease, CPD = Chronic Pulmonary Disease, CHD = Chronic Heart Disease, PaO2 = Arterial Partial Pressure of Oxygen, FiO2 = Fraction of Inspired Oxygen, P/F = PaO2 / FiO2, PEEP = Positive End-Expiratory Pressure, CPAP = Continuous Positive Airway Pressure, ARDS = Acute Respiratory Distress Syndrome

Variable (mean±SD) or (n,%)	Furosemide Group (n=………)	Control Group (n=………..)
Age (years)		
Sex (Females)		
SOFA (Randomization)		
CFS		
BMI		
Comorbidities		
Diabetes		
Hypertension		
CKD		
CLD/Cirrhosis		
CPD		
Heart Failure / CHD		
Immunocompromised		
Malignancy		
Smoker		
ARDS Diagnosis and Precipitating Factors		
PaO2 (mm/Hg)		
FiO2 (decimal)		
P/F Ratio		
PEEP/CPAP (cmH2O)		
Bacterial Pneumonia		
Viral Pneumonia		
Sepsis / Septic Shock		
Trauma-Related		
Transfusion-Related		
Inhalational Injury		
Pancreatitis		
ARDS Severity		
Mild		
Moderate		
Severe		

**Table 2:** Dummy table showing primary and secondary outcomes ICU = Intensive Care Unit, LOS = Length of Stay, P/F = Ratio of Arterial Partial Pressure of Oxygen to Fraction of Inspired Oxygen, VFD = Ventilator-Free Days, CI = Confidence Interval

Variable (mean±SD) or (n,%)	Furosemide Group (n=………)	Control Group (n=………..)	Mean/Percent difference (95% CI; p-value)
ICU Mortality Within 28 Days			
Hospital Mortality			
ICU LOS			
Hospital LOS			
P/F Ratio Change Within 7 Days			
28 Days VFD			
Successful Extubation			
Adverse Events			

## Discussion

This may be the first RCT to explore the effect of nebulized furosemide on the outcomes of critically ill, mechanically ventilated ARDS patients, a hypothesis that stems from furosemide’s anti-inflammatory effects, its availability, direct delivery to the lung tissue, in addition to its wide safety margin, and is supported by the trend of mortality benefit in COVID-19 patients previously reported [12].

Indeed, ARDS is a condition characterized by both high volume and impact. With regards to volume, ARDS involves 10% of all ICU patients and 23% of mechanically ventilated patients, which translates into an incidence of 5.5 cases per ICU bed per year [8]. As for the impact, ARDS was associated with about 35%, 40%, and 45% mortality for mild, moderate, and severe ARDS, respectively, as defined by the P/F ratio [30]. Hence, an intervention with the potential to lower mortality, of any magnitude, is worth exploring.

At the completion of this study, findings will be analyzed to confirm or refute the hypothesis. Our interpretation, conclusion, and recommendations will be presented, accompanied by an objective and transparent discussion of the work’s limitations.

## Conclusions

This RCT tackles an important topic in critical care medicine, which involves a large number of critically ill patients and continues to harbor high mortality despite improvements in management.

The results of the study could provide an additional weapon in the arsenal of ICU management of ARDS and could be built upon to refine and improve the use of nebulized furosemide, with the ultimate aim of faster recovery and lower mortality rate. At the end of the study, the conclusion will be modestly formulated within the boundaries of experimental uncertainty and limitations of the work.
